# Associations of physical activity at work and household income with obesity: a cross-sectional study among rural adults in Korea

**DOI:** 10.4178/epih.e2021003

**Published:** 2020-12-29

**Authors:** Su Yeon Kye, Heeyoun Cho, Tran Thi Phuong Thao, Jin-Kyoung Oh, Min Kyung Lim

**Affiliations:** 1 Division of Cancer Control and Policy, National Cancer Control Institute, National Cancer Center, Goyang, Korea; 2 Department of Cancer Control and Population Health, Graduate School of Cancer Science and Policy, National Cancer Center, Goyang, Korea; 3 Division of Cancer Prevention and Early Detection, National Cancer Control Institute, National Cancer Center, Goyang, Korea

**Keywords:** Exercise, Obesity, Rural population, Adult, Korea

## Abstract

**OBJECTIVES:**

This study was performed to identify the effect of physical activity at work on obesity and to analyze the contribution of socioeconomic factors and health behaviors to this association, which has been relatively little studied.

**METHODS:**

From the Korean National Cancer Center Community Cohort, a total of 5,587 adults (2,125 men; 3,462 women) aged more than 30 years living in rural areas were enrolled. Information on socio-demographic factors, health behaviors, and body mass index was gathered using face-to-face interviews and measurements of height and weight.

**RESULTS:**

Inverse associations were identified between vigorous-intensity physical activity at work and obesity in both men and women, while no association was found between vigorous-intensity physical activity during leisure time and obesity. High household income was independently associated with a lower risk of obesity among those who had low levels of vigorous-intensity physical activity at work. Vigorous physical activity at work showed an inverse association with obesity in rural areas where heavy manual labor is common.

**CONCLUSIONS:**

Our findings indicate the necessity to account for various types of physical activity to improve the assessment and prevention of obesity.

## INTRODUCTION

Evidence has shown that physical activity (PA) is a major determinant of obesity. Therefore, accelerating engagement in PA plays a crucial significant role in fighting the obesity epidemic, especially in modern societies wherein excess energy intake from food or drinks is predominant [1]. Although recommended efforts have been implemented as interventions, the prevalence of obesity has yet to significantly decrease in a single country worldwide [2]. Furthermore, it is predicted to dramatically increase along with the rising global burden of obesity-related diseases in upcoming years [3]. Therefore, more concerted efforts are needed to create PA interventions and to improve obesity prevention strategies globally.

Numerous studies have investigated the role of PA in obesity to suggest appropriate interventions for preventing weight gain and maintaining a healthy weight. However, most researchers have focused on leisure-time PA [4-7] and have not accounted for other domains, including occupational, transportation, and household PA [8,9]. Thus, it is necessary to identify how various patterns of PA affect obesity to obtain a more comprehensive understanding of the association between PA and obesity. In particular, although occupational PA also has a significant impact on total caloric expenditure, few studies have assessed occupational PA together with leisure-time PA in detail [10,11].

People living in developed countries such as Korea have double the risk of developing obesity as those in low-income and middle-income countries [2]. In Korea, the prevalence of obesity dramatically increased from 25% to 43% among men from 1998 to 2018, but stabilized at 26% among women. By 2030, the prevalence of obesity is estimated to reach 62% for men and 37% for women [12,13]. Meanwhile, the frequency of leisure-time PAs was recently found to be very low among the Korean population. In particular, 50% of Koreans reported no weekly moderate-intensity to vigorous-intensity PAs, and only 20% reported participating in such activities 1-2 times/wk [14]. Moreover, most studies have focused on the effects of leisure-time PAs on obesity, while only limited research has explored the various patterns of PA and obesity among more neglected classes of people, including older adults, those living in rural areas, and those with low economic status. Therefore, the present study aimed to investigate the associations of PA during leisure and at work with obesity among adults in rural Korea.

## MATERIALS AND METHODS

### Data source and study population

This study had a cross-sectional design and used baseline survey data from the Korean National Cancer Center (KNCC) Community Cohort, a community-based prospective cohort study designed to investigate the relationships between the risk of cancer and various environmental factors in Korea. This study surveyed 16,304 adults aged 30 years or older between 1993 and 2010 [15]. A total of 5,587 adults (2,125 men; 3,462 women) were recruited to the cohort from July 2003 to July 2006 and were residents of the rural areas included in the final analysis.

### Measures

At the time of enrollment, well-trained interviewers conducted face-to-face interviews with participants using a structured questionnaire that elicited information on various demographic characteristics: gender, age (<50, 50-59, 60-69, or ≥70 years), job (farmer or others), marital status (couple or single), education level (no formal education, primary/middle school, high school, or higher), average household income (1,000 Korean won/mo; < 500, 500- 1,500, or ≥ 1,500), smoking status (non-smokers: < 100 cigarettes during one’s lifetime; past smokers: ≥ 100 cigarettes during one’s lifetime but did not smoke at the time of the interview; current smokers: ≥ 100 cigarettes during one’s lifetime and smoked at the time of interview), and alcohol intake (non-drinkers: 0 g/d; moderate drinkers: < 24 g/d; heavy drinkers: ≥ 24 g/d) [16]. Participants were asked to identify the number of times they participated in vigorous-intensity physical activity during leisure time (VPAL) (e.g., jogging, riding a bike with uphill terrain, tennis, swimming, or aerobic dancing), vigorous-intensity physical activity at work (VPAW) (e.g., carrying a heavy load, digging, rice planting, weeding, or other hard labor), and moderate-intensity physical activity (MPA) (e.g., walking, golf, balling, or riding a bike on flat terrain) during the past year. Frequency was classified as low (< 30 min/wk) and high (≥ 30 min/wk) for VPAL and as low (< 30 min/wk), medium (≥30 min/wk to <1 hr/d), and high (≥ 1 hr/d) for VPAW or MPA [17]. Participants’ height and weight were measured and used to calculate body mass index (BMI; < 23, ≥ 23 to < 25, or ≥ 25 kg/m^2^) [18].

### Statistical analysis

The chi-square test was used to compare the distributions of various characteristics by gender. Multiple logistic regression analysis was used to determine the association between obesity and patterns of PAs adjusting for age, gender, smoking status, alcohol consumption, and household income after excluding job type, marital status, and educational attainment, all of which showed collinearity with household income. The combined effect of PA with other independent variables was analyzed to determine the impact of PA on obesity. Data were analyzed using SAS version 9.2 (SAS Institute Inc., Cary, NC, USA). All statistical significance testing was 2-sided with an α-error of 0.05.

### Ethics statement

The study protocol was approved by the Institutional Review Board (IRB) of the KNCC (IRB No. NCCNHS02-007; NCCNHS03-081-1; NCCNCS-07-080). All study participants signed an informed consent form before inclusion in their respective cohort.

## RESULTS

More than 80% of study participants were older than 50 years and had less than a middle school education. More than half of participants were farmers and had a low household income. The prevalence of current smoking and ever drinking alcohol were 19.1% and 34.5%, respectively. While only 14.9% of participants had high levels of VPAL, 41.0% had high levels of VPAW. The prevalence of obesity was 36.5% and reached 61.4% when overweight participants were included.

Men were more educated and had a higher income than women. Ever smoking, ever drinking alcohol, and higher VPAL and VPAW were more prevalent in men than in women. By contrast, higher MPA was more prevalent in women than in men. In addition, women had a higher prevalence of obesity than men (40.8 vs. 29.3%) (Table 1).

Having higher VPAW showed an inverse association with obesity in both men (low vs. middle: odds ratio [OR], 0.60; 95% confidence interval [CI], 0.45 to 0.81; low vs. high: OR, 0.50; 95% CI, 0.39 to 0.63) and women (low vs. middle: OR, 0.74; 95% CI, 0.59 to 0.92; low vs. high: OR, 0.64; 95% CI, 0.54 to 0.75) after adjusting for other variables as appropriate. However, VPAL and MPA did not show any significant associations with obesity. Age older than 60 years, a low income, and current smoking were associated with obesity in men. Being aged 50-59 years and drinking moderately were associated with obesity in women (Table 2).

Regardless of smoking status, alcohol intake, household income, and the level of VPAL, a higher level of VPAW was a protective factor associated with obesity, and these results remained similar in both genders. In addition, high household income significantly reduced the risk of obesity among women who engaged in low-intensity PA (Table 3).

## DISCUSSION

This is the first study to evaluate the associations of different patterns of PA and obesity among older adults in rural areas. The obesity prevalence in the present study was moderately higher than that in the national data of the same year (36.5 vs. 31.3%). Furthermore, even though the national data indicated a higher obesity prevalence in men than in women (34.7 vs. 27.3%), the present results showed a higher prevalence in women (40.8 vs. 29.3%). The higher prevalence of obesity may be attributed to the older age of most participants, as previous studies have found a relatively high obesity prevalence in this population [19,20]. In general, aging is considered a risk factor for obesity due to excess fat accumulation with lower basal metabolism led by changes in lifestyle with retirement, chronic positive energy balance, and lack of PA associated with age-related diseases. In particular, the hormonal effects caused by menopause could result in greater increases of obesity in women at menopausal age, as suggested by the results of the current study. However, this trend stops or even reverses in those aged more than 60 years, as found in the current study results, with a decrease of obesity prevalence in men after adjusting for the effects of other covariates [21].

One of the major findings of the present study is the lack of a protective effect of VPAL in the study population. This result contrasts with the findings of numerous studies that have confirmed leisure-time PA to be one of the main strategies for obesity prevention [4,22-25]. Interestingly, this difference in the effect of VPAL on obesity may be explained by the population. Most study participants (85.0%) had low levels of leisure-time PA, possibly due to low income and educational levels as well as insufficient facilities for physical exercise in rural communities [26,27]. Instead, the only robust association seen in our study was between VPAW and obesity, regardless of smoking status, alcohol intake, or the level of VPAL. Many studies on the effect of VPAW and obesity have generally shown less consistent results. Some of these studies suggested no associations between VPAW and obesity [19,24,28,29], while others found inverse or positive associations of VPAW with obesity [20,30,31]. Stamatakis et al. [32] reported that domestic activity did not appear to be protective toward obesity, as these types of PAs differ from leisure-time PA. PA during leisure time is characterized by the use of large muscle groups, whereas PA at work mainly utilizes smaller upper-body muscles and is more intermittent, less rhythmic, and often non-locomotory. However, the inverse association observed herein between VPAW and obesity could be explained by the characteristics of the study participants. More than half of the participants were farmers, which is an occupational sector that demands more heavy-intensity work [33]. PA involving heavy-intensity work can reduce the risk of obesity, especially for those who participate in hard manual labor in rural areas [33], as suggested in several previous studies that have reported similar results [20,34]. These findings were consistent after adjusting for other covariates and combined with other potential attributable factors that may be associated with VPAW or obesity. Therefore, the findings provide valuable public health implications for the development and implementation of weight gain prevention strategies, particularly in rural areas where limited attention has been paid to these issues.

Smoking also independently contributed to obesity. In most studies, non-smokers were found have a significantly higher average BMI than smokers [35,36], and our results are in line with this. Nicotine may increase basal metabolism, and smoking cessation could increase weight gain without routine PAs. Compared with non-drinking, moderate (but not heavy) alcohol drinking was associated with a slightly increased prevalence of obesity in the current study. According to our results, 1 or 2 drinks per day decreased the odds of obesity. These results do not match those of other previous studies that suggested an inverse association between increased alcohol consumption and weight gain [37]. Higher alcohol consumption could be a reason for obesity, through the additional calorie intake from alcohol consumption, or a reason for weight loss due to malnutrition caused by inappropriate nutrient and calorie intake among heavy drinkers. Among study participants, the proportion of heavy drinking was low in men and rare in women since drinking is not as socially acceptable for women. Meanwhile, moderate drinking was identified as positively associated with obesity in men. High household income was significantly associated with decreased obesity in men, but only middle income showed a significant positive association with obesity in women. The associations remained still significant after excluding other covariates including VPAW. Possible explanations include the possibility that higher socioeconomic status could allow people to consider and implement a positive lifestyle, including a healthy diet, or lead to excess calorie intake due to misconceptions about appropriate eating. This relationship may also depend on individuals’ access to and understanding of appropriate information for maintaining a healthy weight with social support and relationships to help them, even if these factors were not considered in the present study.

Although the associations of VPAW and household income with obesity in an aged, rural population are novel findings, this study had several limitations. Due to the nature of this cross-sectional study, we could not determine the temporal relationship between VPAW and obesity. Present challenges for clarifying the true impact of PA on weight gain or reduction should continue to be researched in general populations with longitudinal designs, because another study showed evidence that obesity may be a barrier to engaging in PA [38]. In addition, PA is related to both energy expenditure and energy intake, which cannot be easily and accurately measured at the population level. Recalling past PA is a highly complex cognitive task that may not provide accurate estimates in absolute amounts or may have differential outcomes between the young and very old due to the use of ambiguous terms such as “physical activity,” “vigorous intensity,” and “leisure time” [39,40]. Nonetheless, the present study results are unlikely to be distorted by this issue, as a standardized questionnaire was applied to estimate PA and the study population was relatively homogeneous in terms of age, residential area, education, and job. Diet is another important factor associated with obesity that was not measured or considered in the present study. However, it is reasonable to assume that the effects of diet would be the same across the strata of PA and other potential factors, as no reason was found to hypothesize the presence of differences in diet by strata.

There is little question as to the importance of PA for weight management, and our study provides further evidence for the positive effects of vigorous occupational PA on obesity. These results indicate that activity derived from a physically active occupation can reduce obesity, especially for those who participate in hard manual labor in rural areas. Meanwhile, vigorous leisuretime PA did not reduce weight gain. These findings will have important public health implications for the development and implementation of weight gain prevention strategies.

## Figures and Tables

**Table 1.** General characteristics and physical activities of study subjects

Characteristics	Total	Men	Women	p-value
Total	5,587 (100)	2,125 (38.0)	3,462 (62.0)	
Age (yr)				<0.001^1^
<50	1,019 (18.2)	347 (16.3)	672 (19.4)	
50-59	1,273 (22.8)	490 (23.1)	783 (22.6)	
60-69	2,185 (39.1)	815 (38.4)	1,370 (39.6)	
≥70	1,110 (19.9)	473 (22.3)	637 (18.4)	
Job				<0.001^1^
Farmer	2,968 (57.3)	1,301(65.9)	1,667 (52.0)	
Others	2,212 (42.7)	674 (34.1)	1,538 (48.0)	
Marital status				<0.001^1^
Couple	4,154 (76.1)	1,928 (92.4)	2,226 (65.9)	
Single	1,308 (24.0)	158 (7.6)	1,150 (34.1)	
Educational attainment				<0.001^1^
No formal education	855 (25.3)	151 (11.5)	704 (34.0)	
Primary or middle school	1,908 (56.4)	778 (59.1)	1,130 (54.6)	
High school or more	623 (18.4)	388 (29.5)	235 (11.4)	
Household income (103 Korean won/mo)				<0.001^2^
Low (<500)	2,716 (50.9)	872 (42.6)	1,844 (56.1)	
Middle (500-1,500)	1,492 (28.0)	644 (31.5)	848 (25.8)	
High (≥1,500)	1,130 (21.2)	532 (26.0)	598 (18.2)	
Smoking				<0.001^1^
Non-smoker	3,528 (63.9)	398 (18.9)	3,130 (91.8)	
Former smoker	937 (17.0)	831 (39.4)	106 (3.1)	
Current smoker	1,053 (19.1)	881 (41.8)	172 (5.1)	
Alcohol intake (g/d)				<0.001^2^
Non-drinking	3,368 (65.5)	670 (34.3)	2,698 (84.4)	
Moderate drinking (<24)	1,036 (20.1)	597 (30.6)	439 (13.7)	
Heavy drinking (≥24)	742 (14.4)	684 (35.1)	58 (1.8)	
Vigorous-intensity physical activity at leisure time (min/wk)				<0.001^1^
Low (<30)	4,645 (85.1)	1,673 (80.2)	2,972 (88.1)	
High (≥30)	816 (14.9)	414 (19.8)	402 (11.9)	
Vigorous-intensity physical activity at work				<0.001^2^
Low (<30 min/wk)	2,301 (42.1)	683 (32.7)	1,618 (48.0)	
Middle (≥30 min/wk-<1 hr/d)	923 (16.9)	402 (19.3)	521 (15.4)	
High (≥1 hr/d)	2,237 (41.0)	1,002 (48.0)	1,235 (36.6)	
Moderate-intensity physical activity				0.006^2^
Low (<30 min/wk)	1,064 (19.5)	465 (22.3)	599 (17.8)	
Middle (≥30 min/wk-<1 hr/d)	2,658 (48.7)	969 (46.4)	1,689 (50.1)	
High (≥1 hr/d)	1,739 (31.8)	653 (31.3)	1,086 (32.2)	
Body mass index (kg/m^2^)				<0.001^2^
<23	2,140 (38.6)	928 (44.2)	1,212 (35.3)	
23-25	1,380 (24.9)	558 (26.6)	822 (23.9)	
≥25	2,020 (36.5)	616 (29.3)	1,404 (40.8)	

Values are presented as number (%).

1 Chi-square test.

2 Mantel-Haenszel chi-square test.

**Table 2.** Adjusted ORs and 95% CIs of obesity (BMI≥25 kg/m^2^) by general characteristics and physical activities

Characteristics		Total			Men			Women		
		Prevalence	Adjusted OR^1^ (95% CI)	Adjusted OR^2^ (95% CI)	Prevalence	Adjusted OR^1^ (95% CI)	Adjusted OR^2^ (95% CI)	Prevalence	Adjusted OR^1^ (95% CI)	Adjusted OR^2^ (95% CI)
		% (n/N)			% (n/N)			% (n/N)		
Age (yr)										
	<50	37.6 (376/1,001)	1.00 (reference)	1.00 (reference)	39.2 (133/339)	1.00 (reference)	1.00 (reference)	36.7 (243/662)	1.00 (reference)	1.00 (reference)
	50-59	44.6 (565/1,267)	1.38 (1.16, 1.63)	1.52 (1.26, 1.84)	37.1 (181/488)	0.91 (0.69, 1.21)	1.03 (0.75, 1.40)	49.3 (384/779)	1.68 (1.36, 2.07)	1.71 (1.34, 2.17)
	60-69	35.4 (771/2,176)	0.93 (0.79, 1.08)	1.00 (0.82, 1.21)	26.8 (217/811)	0.57 (0.43, 0.74)	0.59 (0.43, 0.81)	40.6 (554/1,365)	1.18 (0.97, 1.43)	1.21 (0.95, 1.54)
	≥70	28.1 (308/1,096)	0.67 (0.56, 0.81)	0.70 (0.56, 0.89)	18.3 (85/464)	0.35 (0.25, 0.48)	0.35 (0.23, 0.52)	35.3 (223/632)	0.94 (0.75, 1.18)	0.96 (0.72, 1.29)
Smoking										
	Non-smoker	40.6 (1,423/3,503)	1.00 (reference)	1.00 (reference)	34.8 (137/394)	1.00 (reference)	1.00 (reference)	41.4 (1,286/3,109)	1.00 (reference)	1.00 (reference)
	Former smoker	32.8 (304/927)	0.98 (0.80, 1.20)	0.94 (0.75,1.17)	32.6 (268/821)	0.98 (0.76, 1.28)	0.92 (0.69, 1.21)	34.0 (36/106)	0.80 (0.53, 1.21)	0.84 (0.53, 1.31)
	Current smoker	25.5 (266/1045)	0.64 (0.52, 0.78)	0.64 (0.52, 0.80)	24.1 (211/875)	0.56 (0.43, 0.73)	0.58 (0.44, 0.77)	32.4 (55/170)	0.72 (0.52, 1.01)	0.70 (0.48, 1.01)
Alcohol intake (g/d)										
	Non-drinking	37.3 (1,247/3,344)	1.00 (reference)	1.00 (reference)	25.6 (170/664)	1.00 (reference)	1.00 (reference)	40.2 (1,077/2,680)	1.00 (reference)	1.00 (reference)
	Moderate drinking (<24)	37.9 (388/1,025)	1.21 (1.03, 1.41)	1.25 (1.06, 1.48)	35.0 (206/589)	1.41 (1.10, 1.81)	1.47 (1.13, 1.90)	41.7 (182/436)	1.09 (0.88, 1.34)	1.12 (0.90, 1.39)
	Heavy drinking (≥24)	27.8 (205/738)	0.91 (0.74, 1.11)	0.98 (0.79, 1.22)	27.4 (186/680)	0.99 (0.77, 1.27)	1.09 (0.84, 1.41)	32.8 (19/58)	0.75 (0.43, 1.31)	0.85 (0.48, 1.51)
Household income (103 KRW/mo)										
	Low (<500)	33.7 (911/2,707)	1.00 (reference)	1.00 (reference)	23.1 (200/867)	1.00 (reference)	1.00 (reference)	38.6 (711/1,840)	1.00 (reference)	1.00 (reference)
	Middle (500-1,500)	38.8 (570/1,471)	1.17 (1.01, 1.35)	1.17 (1.01, 1.37)	29.8 (188/632)	1.14 (0.90, 1.46)	1.23 (0.95, 1.60)	45.5 (382/839)	1.25 (1.04, 1.49)	1.22 (1.01, 1.47)
	High (≥1,500)	38.4 (429/1,118)	1.14 (0.96, 1.35)	1.06 (0.89, 1.27)	38.8 (205/529)	1.46 (1.12, 1.90)	1.34 (1.00, 1.78)	38.0 (224/589)	0.97 (0.77, 1.21)	0.90 (0.71, 1.15)
Vigorous-intensity physical activity at leisure time (min/wk)										
	Low (<30)	35.8 (1,653/4,613)	1.00 (reference)	1.00 (reference)	28.3 (469/1,658)	1.00 (reference)	1.00 (reference)	40.1 (1,184/2,955)	1.00 (reference)	1.00 (reference)
	High (≥30)	38.2 (310/811)	1.12 (0.95, 1.31)	1.12 (0.94, 1.33)	32.9 (135/411)	1.08 (0.85, 1.37)	0.99 (0.76, 1.30)	43.8 (175/400)	1.15 (0.93, 1.43)	1.20 (0.95, 1.52)
Vigorous-intensity physical activities at work										
	Low (<30 min/wk)	42.6 (971/2,277)	1.00 (reference)	1.00 (reference)	37.2 (251/675)	1.00 (reference)	1.00 (reference)	44.9 (720/1,602)	1.00 (reference)	1.00 (reference)
	Middle (≥30 min/wk-<1 hr/d)	33.4 (307/919)	0.67 (0.57, 0.79)	0.70 (0.59, 0.83)	27.2 (109/400)	0.57 (0.43, 0.75)	0.60 (0.45, 0.81)	38.1 (198/519)	0.71 (0.58, 0.88)	0.74 (0.59, 0.92)
	High (≥1 hr/d)	30.7 (685/2,228)	0.58 (0.52, 0.66)	0.59 (0.51, 0.68)	24.5 (244/994)	0.50 (0.40, 0.62)	0.50 (0.39, 0.63)	35.7 (441/1,234)	0.63 (0.54, 0.73)	0.64 (0.54, 0.75)
Moderate-intensity physical activity										
	Low (<30 min/wk)	36.5 (385/1,054)	1.00 (reference)	1.00 (reference)	27.9 (128/458)	1.00 (reference)	1.00 (reference)	43.1 (257/596)	1.00 (reference)	1.00 (reference)
	Middle (≥30 min/wk-<1 hr/d)	35.9 (947/2,636)	0.92 (0.79, 1.07)	0.98 (0.83, 1.16)	28.7 (276/960)	1.04 (0.81, 1.33)	1.04 (0.79, 1.37)	40.0 (671/1,676)	0.87 (0.72, 1.05)	0.94 (0.77, 1.16)
	High (≥1 hr/d)	36.4 (631/1,734)	0.94 (0.80, 1.10)	0.96 (0.80, 1.14)	30.7 (200/651)	1.18 (0.91, 1.55)	1.19 (0.88, 1.60)	39.8 (431/1,083)	0.85 (0.69, 1.04)	0.87 (0.70, 1.08)

OR, odds ratio; CI, confidence interval; BMI, body mass index; KRW, Korean won.

1 Adjusted for age and gender.

2 Adjusted for age, gender, smoking, alcohol, income, vigorous-intensity physical activity at leisure time, vigorous-intensity physical activities at work, and moderate-intensity physical activity.

**Table 3.** Adjusted ORs and 95% CIs of obesity (BMI>25 kg/m^2^) according to the combination of VPAW with smoking, alcohol intake, household income, and vigorous-intensity recreational physical activities

Variables		Total		Men		Women	
		Adjusted OR^1^ (95% CI)	Adjusted OR^2^ (95% CI)	Adjusted OR^1^ (95% CI)	Adjusted OR^2^ (95% CI)	Adjusted OR^1^ (95% CI)	Adjusted OR^2^ (95% CI)
VPAW/Smoking status^3^							
	Low/Non-smoker	1.00 (reference)	1.00 (reference)	1.00 (reference)	1.00 (reference)	1.00 (reference)	1.00 (reference)
	Low/Current smoker	0.79 (0.61, 1.01)	0.85 (0.65, 1.12)	0.69 (0.49, 0.96)	0.80(0.55, 1.16)	0.78(0.50, 1.22)	0.77 (0.47,1.26)
	Middle+High/Non-smoker	0.64 (0.57, 0.73)	0.66 (0.58, 0.76)	0.59 (0.45, 0.76)	0.61(0.46, 0.81)	0.65(0.57, 0.76)	0.67 (0.57, 0.78)
	Middle+High/Current smoker	0.37 (0.30, 0.46)	0.37 (0.29, 0.48)	0.31 (0.23, 0.41)	0.33(0.24, 0.44)	0.44(0.26, 0.74)	0.43 (0.25, 0.75)
VPAW/Alcohol intake (g/d)							
	Low/Non-drinking	1.00 (reference)	1.00 (reference)	1.00 (reference)	1.00 (reference)	1.00 (reference)	1.00 (reference)
	Low/Moderate drinking (<24)	1.14 (0.90, 1.45)	1.20 (0.94, 1.53)	1.45 (0.96, 2.17)	1.57 (1.03, 2.39)	0.96 (0.70, 1.32)	1.00 (0.72, 1.39)
	Low/Heavy drinking (≥24)	1.16 (0.84, 1.61)	1.21 (0.87, 1.69)	1.21 (0.80, 1.84)	1.28 (0.83, 1.99)	0.99 (0.44, 2.22)	1.08 (0.47, 2.46)
	Middle+High/Non-drinking	0.63 (0.55, 0.73)	0.63 (0.55, 0.74)	0.56 (0.39, 0.80)	0.59 (0.41, 0.87)	0.66 (0.56, 0.77)	0.65 (0.55, 0.77)
	Middle+High/Moderate drinking (<24)	0.82 (0.67, 1.01)	0.82 (0.66, 1.01)	0.83 (0.58, 1.18)	0.84 (0.58, 1.22)	0.80 (0.60, 1.06)	0.79 (0.59, 1.06)
	Middle+High/Heavy drinking (≥24)	0.54 (0.41, 0.69)	0.56 (0.43, 0.73)	0.54 (0.38, 0.77)	0.58 (0.40, 0.85)	0.42 (0.19, 0.94)	0.44 (0.19, 1.00)
VPAW4/Household income (103 KRW/mo)							
	Low/Low (<500)	1.00 (reference)	1.00 (reference)	1.00 (reference)	1.00 (reference)	1.00 (reference)	1.00 (reference)
	Low/Middle (500-1,500)	1.23 (0.99, 1.53)	1.26 (1.00, 1.59)	1.36 (0.90, 2.05)	1.58 (1.02, 2.46)	1.24 (0.96, 1.61)	1.23 (0.93, 1.63)
	Low/High (≥1,500)	0.75 (0.59, 0.96)	0.74 (0.58, 0.96)	1.21 (0.80, 1.81)	1.30 (0.84, 2.01)	0.61 (0.45, 0.83)	0.57 (0.41, 0.78)
	Middle+High/Low (<500)	0.52 (0.44, 0.62)	0.55 (0.46, 0.66)	0.49 (0.35, 0.68)	0.57 (0.40, 0.82)	0.55 (0.45, 0.67)	0.57 (0.46, 0.69)
	Middle+High/Middle (500-1,500)	0.62 (0.51, 0.75)	0.63 (0.51, 0.77)	0.57 (0.41, 0.8)	0.63 (0.44, 0.91)	0.69 (0.54, 0.88)	0.68 (0.53, 0.87)
	Middle+High/High (≥1,500)	0.77 (0.62, 0.97)	0.78 (0.62, 0.99)	0.75 (0.52, 1.08)	0.79 (0.53, 1.16)	0.80 (0.59, 1.08)	0.80 (0.59, 1.10)
VPAW^4^/VPAL^5^							
	Low/Low	1.00 (reference)	1.00 (reference)	1.00 (reference)	1.00 (reference)	1.00 (reference)	1.00 (reference)
	Low/High	0.84 (0.65, 1.09)	0.81 (0.61, 1.07)	0.71 (0.47, 1.08)	0.69 (0.44, 1.10)	0.89 (0.64, 1.25)	0.83 (0.58, 1.20)
	Middle+High/Low	0.56 (0.5, 0.64)	0.58 (0.51, 0.66)	0.45 (0.36, 0.57)	0.48 (0.37, 0.61)	0.61 (0.53, 0.71)	0.62 (0.53, 0.73)
	Middle+High/High	0.77 (0.63, 0.95)	0.78 (0.63, 0.97)	0.61 (0.44, 0.83)	0.58 (0.41, 0.81)	0.89 (0.67, 1.18)	0.94 (0.70, 1.25)

OR, odds ratio; CI, confidence interval; BMI, body mass index; VPAW, vigorous-intensity physical activity at work; KRW, Korean won; VPAL, vigorous-intensity physical activity during leisure time.

1 Adjusted for age and gender.

2 Adjusted for age, gender, smoking, alcohol intake, and household income.

3 Non-smoker: non-smokers and former smokers.

4 VPAW: low (<30 min/wk), middle (≥30 min/wk-<1 hr/d), high (≥1 hr/d).

5 VPAL: low (<30 min/wk), high (≥30 min/wk).
